# Dietary assessment methods in epidemiologic studies

**DOI:** 10.4178/epih/e2014009

**Published:** 2014-07-22

**Authors:** Jee-Seon Shim, Kyungwon Oh, Hyeon Chang Kim

**Affiliations:** 1 Cardiovascular and Metabolic Diseases Etiology Research Center, Yonsei University College of Medicine, Seoul, Korea; 2 Division of Health and Nutrition Survey, Korea Centers for Disease Control and Prevention, Osong, Korea; 3 Department of Preventive Medicine, Yonsei University College of Medicine, Seoul, Korea

**Keywords:** Dietary assessment, Food frequency questionnaire, 24-hour dietary recall, Dietary record

## Abstract

Diet is a major lifestyle-related risk factor of various chronic diseases. Dietary intake can be assessed by subjective report and objective observation. Subjective assessment is possible using open-ended surveys such as dietary recalls or records, or using closed-ended surveys including food frequency questionnaires. Each method has inherent strengths and limitations. Continued efforts to improve the accuracy of dietary intake assessment and enhance its feasibility in epidemiological studies have been made. This article reviews common dietary assessment methods and their feasibility in epidemiological studies.

## INTRODUCTION

Diet is a major lifestyle-related risk factor of a wide range of chronic diseases. Changes in dietary habits have been found to reduce cancer incidence by one-third [1]. Dietary information has been useful in cardiovascular disease risk prediction [2] and consuming a nutrient-dense diet was associated with a low risk of all-cause mortality [3]. Contrary to other lifestyle risk factors (e.g., smoking), dietary exposures are very difficult to measure because all individuals eat foods, even if the amount and the kind of food consumed is various between subjects, and people rarely perceive what they eat and how much they do [4]. Inaccurate dietary assessment may be a serious obstacle of understanding the impact of dietary factors on disease.

Specific biochemical markers have been used as a surrogate to measure the dietary intake of selected nutrients or dietary components in epidemiological studies [5-7]. Previous studies have found these markers to be highly correlated with dietary intake levels, free of a social desirability bias, independent of memory, and not based on subjects' ability to describe the type and quantity of food consumed [8]. Thus, these biochemical markers may provide more accurate measures than dietary intake estimates do. However, a number of biomarkers have been known to provide integrated measures reflecting their absorption and metabolism after consumption, and they are also affected by disease or homeostatic regulation, thus their values cannot be translated into the subject's absolute dietary intake [9]. Moreover, the results based on biomarkers cannot provide dietary recommendations to modify a subject's dietary habit. Thus, direct assessment of dietary intake may be more informative than biomarkers are [8,10].

Among the available dietary assessment methods, the food frequency questionnaire (FFQ) has been widely used in large epidemiological studies since the 1990s. After doubts of their accuracy were raised in the 2000s [11,12], numerous changes to the assessment methods have been made. Some researchers have shifted their focus and concentrated their efforts to improve the feasibility and accuracy of open-ended dietary assessment methods rather than improve the FFQ or further find relevant biomarkers. Other researchers have concentrated their efforts to enhance the accuracy of the FFQ. Assessing dietary exposure accurately with limited resources remains a challenge for researchers. Thus, we aimed to review common methods for dietary assessment and their feasibility in epidemiological studies.

## DIETARY ASSESSMENT BY OBJECTIVE OBSERVATION

Table 1 summarizes the available dietary assessment methods, including the methods, collected data, strengths, and limitations considering a conservative approach. Dietary intake can be assessed by objective observation using a duplicate diet approach or food consumption record by a trained research staff. The duplicate diet method collects duplicate samples of a subject's normal diet, and then analyzes it to estimate potential dietary exposures. This method has been mainly used to measure exposure to environmental contaminants such as phthalates and polycyclic aromatic hydrocarbons in foods and beverages [13]. Food consumption record collects dietary information on subjects' food preparation and consumption in their home with the objective observation of skilled field workers. This method is useful in developing countries, especially among those with a low literacy rate or those who prepare a substantial portion of their food at home. In South Korea, the National Nutrition Survey had surveyed households by this direct method to monitor national food consumption from 1969 to 1995 [14]. Well-trained staffs observed and recoded all foods prepared and consumed in the surveyed household for two consecutive days. All data were collected at the household level, thus no information on how foods were consumed by each individual within household or about foods consumed outside the home were collected. Thus, each individual's consumption was indirectly estimated using data on the number, age, and sex of residents in each household sharing the recorded food [15]. With improvement in economic status, increase in eating-out, and advancement in the individual dietary assessment techniques, assessment at the individual level has become widespread in nutritional epidemiological settings.

## DIETARY ASSESSMENT BY SUBJECTIVE REPORT

Subjective dietary assessment methods that assess an individual's intake include the 24-hour dietary recall (24HR), dietary record (DR), dietary history, and FFQ. Data are collected with the help of a trained interviewer or by self-report.

### Twenty-four-hour dietary recall and dietary record in a conservative approach

The 24HR and DR are completely open-ended surveys and collect a variety of detailed information about food consumed over a specific period. The 24HR is conducted in an in-depth interview manner and typically requires 20 to 30 minutes to complete a single day recall. Detailed data about food preparation methods, ingredients used in mixed dishes, and the brand name of commercial products may be required according to the research question. The amounts of each food consumed are estimated in reference to a common size container (e.g., bowls, cups, and glasses), standard measuring cups and spoons, a three-dimensional food model, or two-dimensional aids such as photographs. One advantage of the 24HR is that a relatively minimal burden is imposed on respondents. However, an inevitable limitation is that all information depends on the respondents' memory and the skills of a well-trained interviewer to minimize recall bias. Conversely, DR collects data by subjects' self-record at the time the food are eaten, thus minimizes reliance on a subjects' memory. To obtain accurate data, however, respondents must be trained before participating the survey. Therefore, a high level of motivation is required and relatively large burden is passed onto the respondents [4,15].

Both methods have a few common strengths. Both use open-ended questions so that abundant information can be collected and analyzed in various aspects. In addition, both methods can be easily applied to diverse groups with a wide range of eating habits and may be used to estimate the average intake of a certain population. In many countries including South Korea, the 24HR is the most commonly used in national surveys [16], and both methods are also frequently applied to randomized clinical trials and cohort studies [17,18]. However, these methods have limitations when used to study chronic diseases, a major public health concern. One limitation is that both methods are mainly focused on short-term intake, but long-term dietary exposure is especially of interest when investigating chronic diseases. Thus, to measure average intake, multiple 24HRs or DRs are needed. Repeated measurement not only requires a lot of resources and time but survey repetition can also influence a respondents' diet. Previous studies have found some respondents may improve their dietary habits unintentionally through self-reflection. However, some respondents may alter their diet intentionally to avoid a burden on responses or even choose to not report actual intake [4,15]. Another limitation spawns from the open-ended format that requires considerable efforts in the course of data collection, entry, and analyses. Each questionnaire requires careful review by the research staff to ensure that all reported data are included. After initial review, all foods and mixed dishes consumed according to the detailed descriptions of the respondents should be matched and coded with the most appropriate food listed in the food composition database. Moreover, the quantity of food consumed should be converted to its actual weights. When the reported information is changed to the corresponding food code and weight, actual intakes can be calculated. These processes tend to be time-consuming, laborious, and highly expensive to implement.

### Twenty-four-hour dietary recall and dietary record with newer technologies

Despite the aforementioned limitations, multiple 24HRs and DRs have inherent strengths in etiologic studies of chronic diseases. First, both methods collect actual intake on specific days. Second, the burden of memory may be less for these methods than that of the FFQ, which requires recall over a long period (e.g., the previous 12 months). Last, usual intake can also be estimated if repeated. Owing to these strengths, innovative technologies focusing on reducing the respondents' burden, improving accuracy, and making multiple self-administrations possible have been recently incorporated to improve their feasibility in epidemiological studies. Recently, several reports have discussed their use and implications in clinical and research settings [19-21].

Although many techniques are still under development, major advances have been made. Interactive computer-based technologies, which were introduced relatively early in dietary assessment method development, aims to be a comprehensive system for data collection, coding, entry, and calculation of intakes. Examples includes the Automated Multiple Pass Method (AMPM) for administering the 24HR in the US National Health and Nutrition Examination Survey [22] and a menu-driven standardized 24HR program (called the EPIC-Soft) in the European Prospective Investigation into Cancer and Nutrition study [23] that allow interviewers to collect, probe, and identify reported intake in a standardized manner, thus improving the accuracy of the data, even if they are used in diverse populations. Having limitations in time, location, and the number of interviewers available for each study, these technologies remain relatively costly for implementation in large-scale epidemiological studies.

Considerably overlapped with the computer-based approach, web-based technologies enable researchers to collect data regardless of a time and a location, assuming internet access is available. Recently the National Cancer Institute in the US. has developed an internet-based technique, called the Automated Self-Administered 24HR that is based on the AMPM approach [24]. This internet-based technique includes an online tutorial, digital images for food identification and portion-size estimation, and various audio files. Thus, those with low literacy can easily complete the survey, and researchers can collect real-time data. Other internet-based technologies designed for face-to-face, standardized interview administration have been developed, such as the Diet Evaluation System (DES) that was developed in South Korea [25].

In addition, mobile phone applications that allow users to enter dietary intake data have been released. Subjects can manually record their diet by choosing corresponding items from a pre-defined list of foods and beverages, and the quantity of food consumed can be recorded by selecting from pre-defined portion sizes [26]. In South Korea, SmartDiet is an application that was developed for dietary management and education, and this application have been evaluated for their effectiveness and feasibility in clinical settings [27]. Multiple functions embedded in a mobile device can be used to collect data. In Japan, the mobile phone application (called Wellnavi) uses the subject's camera and mobile phone card to report everything that was consumed by sending images before and after eating to the study dietitian [28]. In addition, voice recording such as the Spoken Diet Records has been used to collect data [29]. In Australia, Nutricam allows subjects to capture an image of foods and drinks before consumption and verbally describe the items in the image [30]. Then, subjects upload both the image and voice file to a website for analysis [30]. Recently a wearable electronic device that resembles a necklace includes a camera, microphone, and several other sensors has been introduced [31]. This technology uses the video recording to collect dietary information, and the software identifies eating episode and estimates the amount consumed in the video file. Then, final dietary intakes are calculated automatically. This method is likely to minimize the burden of the subjects using objective observation; however, the technology is still in the experiment stage for using in researches.

Most state-of-the-art technologies must give enormous potentials to be adapted as a major dietary assessment tool in various epidemiological studies to the conservative open-ended methods depending on paper and pencil surveys [19,20,24,32,33]. Table 2 summarizes the strengths and limitations of dietary assessment methods with newer techniques. Software development and the required hardware need high costs in the early stage of the research. However, only if they are prepared, DRs and 24HRs with innovative technologies may reduce their costs and resources for organizing study as well as collecting and handling data, improve consistency of data, collect data in real time and calculate dietary intakes automatically, and allow respondents to focus on dietary assessment [20,23,25,32,33]. While the feasibility of multiple 24HRs and DRs in epidemiological studies has considerably improved with the help of these new technologies, there are still some limitations. First, these methods may be difficult to apply to certain populations who are not familiar with innovative technologies or new devices [32]: Training subjects on how to use these technologies and use a computer including accessing the internet is also required [25]. Furthermore, technical problems in data transfer, storage, battery life, and other concerns must be improved [31]. Most importantly, these new methods do not seem to overcome the methodological problems related to self-report. A previous report found that subjects still had difficulties in recalling and reporting their diet, underreported in repeated assessments, and altered food intake when they knew the survey date in advance [19]. For these reasons, open-ended methods with new technologies have not yet been widely implemented as the primary assessment tool in epidemiological studies.

### Dietary history

To assess individual long-term dietary intake, Burke [34] developed a dietary history method in 1947. This method requires that subjects complete a 24HR, 3-day food diary, and checklist of foods usually consumed. Highly skilled professionals are required to collect information on the participant's usual diet using an in-depth interview (approximately 90 minutes to complete). Thus, this method is rarely used in epidemiological studies.

### Food frequency questionnaire

The FFQ is an advanced form of the checklist in dietary history method, and asks respondents how often and how much food they ate over a specific period [4]. Presenting about 100 to 150 foods, this questionnaire takes 20-30 minutes to complete and can self-administered or collected via interview. This method enables the assessment of long-term dietary intakes in a relatively simple, cost-effective, and time-efficient manner. Thus, various FFQs have been widely employed as a practical instrument since the 1990s [35-37]. FFQs should be developed specifically for each study group and research purposes because diet may be influenced by ethnicity, culture, an individual's preference, economic status, etc. [38]. In South Korea, approximately 20 FFQs have been developed and used in epidemiological studies.

In South Korea, the first FFQ was developed through modification of the FFQs used in Western countries to meet Korean diet characteristics [39]. After, some FFQs were developed following the opinion of experienced dietitians and epidemiologists based on the nutrient contents in Korean food and the results of previous studies [40-42]. Recent FFQs have been developed in a more sophisticated way using actual dietary data collected by the open-ended surveys. Among the various foods consumed by subjects, informative foods are selected according to the extent to which the foods contribute specific nutrients intakes or the extent that the foods explained between-persons variations [43-47]. Then the selected foods are grouped by their nutritional contents or cooking methods, and finally presented in a closed-ended format.

According to the interests of the researchers, FFQs may focus on the intake of specific nutrients [48,49], dietary exposures related to a certain disease [43], or comprehensively assess various nutrients [44,46,47]. In prospective studies, comprehensive assessment is generally recommended because it enables us to assess any dietary components, which were not important at the beginning of a study but might emerge as an important factor later. Comprehensive assessment also enables us to estimate the intakes of various dietary components that might act as a confounder in relation to a key dietary factor and diseases, which allows for statistical adjustment.

According to the way which informative foods present in FFQs, food-based FFQs [16,46,47] such as the Harvard FFQ [50,51] and dish-based FFQs [43-45,52,53] have been developed. Korean and Asian food mainly contains many mixed dishes that are cooked with individual ingredient foods, seasonings, and cooking oils. Thus, food-based FFQs may raise subjects' burden and increase response error, when their subjects do not typically cook their food or are unaware of the ingredients. Moreover, the food-based FFQ [54] tends to underestimate dietary intake more than the dish-based FFQs do [44] because various seasonings (e.g., salt, soy sauce, red pepper paste, soybean paste, etc.) and cooking oils which are highly contributing to the nutrients (e.g., energy, fat, sodium, and β-carotene intake, etc.) intakes are not considered in dietary intake calculations [55,56]. Therefore, the dish-based approach has been recommended as a new strategy to improve dietary assessment in countries with an Asian diet [57-59].

Average consumption frequency can be assessed using open-ended questions [41], but most FFQs collect data across nine possible responses from never to three or more times per day. Various answer choices have been used to improve data quality and reduce the burden on the subjects [60]. For foods eaten seasonally, subjects are typically asked how frequently and over what duration they ate these seasonal foods [42,44,47]. For frequently consumed foods such as coffee, answers are collected directly as an open-ended question in some FFQs [44,61,62].

The utility of questions in FFQs about portion size has been controversial [4]: Some researchers reported that between-person variations in portion size were not important because that variation tends to be smaller than the variation in frequency of consumption [63]. In South Korea, however, data on the portion size of some foods seems to be important, such as cooked rice, because between-person variations might be highly explained by the portion size rather than the frequency [64]. Until now, semi-quantitative FFQs collecting data on the average portion sizes in a closed format have been more widely used in epidemiological studies [39,40,42-49,52,53,61,65-69] than has been the simple FFQs which solely asks about the frequency [16,70] or quantitative FFQs which queries about the amount of food consumption using completely open-ended questions [41], respectively.

FFQs, which use a closed format, should be evaluated for their accuracy before being used as a dietary assessment tool in studies. A correlation coefficients ranging from 0.5 to 0.7 is considered moderate [4]; however, most FFQs from Asian countries including South Korea tend to have correlation coefficients ranging from 0.3 to 0.5 [40-43,61,66,67,71], which is lower than that from Western countries.

Some researchers questioned the value of using FFQs in epidemiological studies [11,12], and this topic continues to be highly debated [57,72-76]. In addition, concentrated efforts to assess usual dietary intakes accurately using FFQs as well as multiple 24HRs or DRs have been made. Newer techniques introduced FFQs that can be optically scanned, perform complex skip algorithms and probe multiple details, and range checks as well as allows for the presentation of pictures of foods for ease in reporting portion sizes. All of these efforts improve the quality of dietary data and enhance our capability to collect complex information.

## CONCLUSION

Dietary intake is difficult to measure, and any single method cannot assess dietary exposure perfectly. Nutritional biomarkers are valid for objective estimates of dietary exposures in anthropometric and clinical assessment, while the 24HR, DR, dietary history, and FFQ are subjective estimates. Numerous efforts have made progress in the accuracy of dietary intake assessment methods, thus the feasibility of open-ended methods with various innovative technologies in epidemiological studies has been substantially enhanced. However, new methods needs higher costs than the FFQs, and intrinsic problems related to self-report remain unsolved. Notwithstanding the discussed limitations, FFQs are still widely used as the primary dietary assessment tool in epidemiological studies.

Recently, it has been suggested that a combination of methods, such as the FFQ with DRs (or 24HR) or the FFQ with biomarker levels, be used to obtain more accurate estimates of dietary intakes than that of individual methods. Considerable efforts to improve the accuracy and feasibility of large epidemiological studies are still ongoing.

In summary, dietary assessment methods should be selected with caution and while considering the research objective, hypothesis, design, and available resources.

## Figures and Tables

**Table 1.** Dietary assessment methods in epidemiological studies

	Duplicate diet approach	Food consumption record	24-Hour dietary recall	Dietary record	Dietary history	Food frequency questionnaire
Methods	Collection of duplicate diet sample and direct analysis	Objective observation by trained staff at the household level	Subjective measure using open-ended questionnaires administered by a trained interviewer	Subjective measure using open-ended, self-administered questionnaires	Subjective measures using open- and closed-ended questionnaires administered by a trained interviewer	Subjective measure using a predefined, self- or interviewer-administered format
Collected date	Actual intake information throughout a specific period	Actual intake information throughout a specific period	Actual intake information over the previous 24 hours	Actual intake information throughout a specific period	Usual intake estimates over a relatively long period	Usual intake estimates over a relatively long period (e.g., 6 months or 1 year)
Strengths	Measurement of dietary exposures possible (e.g., envi ronmental contaminants)	Ease of application among those with low literacy or those who prepare most meals at home	Provides detailed intake data; relatively small respondent burden (literacy not required)	Provides detailed intake data; no interviewer required; no recall bias	Assesses usual dietary intake	Assesses usual dietary intake simply; cost-effective and time-saving; suitable for epidemiological studies
Limitations	Not suitable for large-scale studies	Individual dietary consumption not accurate; Not suitable among those frequently eat outside the home	Possible recall bias; trained interviewer required; possible interviewer bias; expensive and time-consuming; multiple days required to assess usual intake; possible changes to diet if repeated measures	Relatively large respondent burden (literacy and high motivation required, possible under-reporting); expensive and time-consuming; multiple days required to assess usual intake; possible changes to diet if repeated measures	High cost and time-consuming; not suitable for epidemiological studies	Specific to study groups and research aims; uses a closed-ended questionnaire; low accuracy (recall bias); requires accurate evaluation of developed questionnaires

**Table 2.** Strengths and limitations of new techniques in dietary assessment

	24-hour dietary recall	Dietary record	Food frequency questionnaire
Required technology	Software, internet, etc.	Software, internet, PDA, mobile phone, application, etc.	Skip algorithms, questions that ask for multiple details, pictures of foods, etc.
Strengths	Standardized data collection possible (reducing interviewer bias); likely reduce time and cost; improve feasibility	Standardized, real-time data collection possible; likely reduce time and cost; improve feasibility	Able to collect complex information and highly accurate data
Limitations	Inherent bias related to self-report	Inherent bias related to self-report; requires participant training on how to use the technology	Measurement errors related to methodology remain
